# Sunlight in Stables

**Published:** 1885-12

**Authors:** 


### SUNLIGHT IN STABLES.

We tried an experiment some years since to test the effect of absence of light upon on a calf. We had two deep-red calves of the same age (60 days), one weighing 180 pounds and the other 182 pounds. The latter we placed in a dark room, with a trough that could be filled by a spout through a partition. The other was confinen in the same amount of space, but in full light, and both were fed exactly alike for the next three months. The object was to test the effect of light upon such a growing animal. At the end of the time the one in the light weighed 430 pounds and the one in the dark weighed 360 pounds, and its color had faded to a very pale, dirty red. Its eyes were so much affected when admitted to the light that it kept them closed most of the time for the first week or two. The two calves were kept on together, but the one from the dark room never fully recovered from this three months of darkness. It never recovered its bright red color, although the color improved. Any one who noted these two calves during this experiment would never after doubt the impolicy of a dark stable. Sunlight is indispensable to healthy vegetable and animal life. A farmer sees his cat and dog select a belt of sunlight to lie and bask in ; and if he will watch his cattle when turned out he will find them seeking at once the sunny side of the barnyard. And with all these indications before his eyes, still the farmer keeps his animals in a dark stable, much to their discomfort and his pecuniary loss. We do not, of course, include all farmers in this statement, for a small minority fully understand the importance of sunlight in stables, and make ample provision for its introduction.—*Live Stock Journal.*

